# Preservation of renal function in cardiac surgery patients with low cardiac output syndrome: levosimendan vs beta agonists

**DOI:** 10.1186/s12871-019-0888-2

**Published:** 2019-11-17

**Authors:** Jose Luis Guerrero Orriach, I. Navarro Arce, P. Hernandez Rodriguez, A. Raigón Ponferrada, A. Malo Manso, M. Ramirez Aliaga, A. Ramirez Fernandez, J. J. Escalona Belmonte, I. Bellido Estevez, A. Gomez Luque, R. Barrera Serrano, C. S. Toledo Medina, M. Rubio Navarro, J. Cruz Mañas

**Affiliations:** 1 Institute of Biomedical Research in Malaga [IBIMA], Malaga, Spain; 2 Department of Anaesthesiology, Virgen de la Victoria University Hospital, University Campus Teatinos, C.P. 29010 Malaga, Spain; 3 Department of Pharmacology and Pediatrics, School of Medicine, University of Malaga, Malaga, Spain

**Keywords:** Levosimendan, Low cardiac output, Kidney, Cardiac surgery

## Abstract

**Background:**

Some studies have been performed to assess the effects of levosimendan on cardiac function when administered to cardiac surgery patients with low cardiac output syndrome (LCOS) in the immediate postoperative period. Levosimendan is an inotropic agent for the treatment of low cardiac output syndrome that seems to have a protective effect on renal function.

**Methods:**

It is a quasi-experimental study. A total of 100 patients with LCOS received either beta-agonists or levosimendan. We assessed the incidence of postoperative kidney failure in cardiac surgery patients. In patients who had kidney failure at diagnosis of LCOS, we examined whether differences existed in the evolution of kidney failure based on the treatment administered for LCOS. The parameters measured included haemodynamics, oxygen supply, and renal function as assessed by the AKI scale. ANOVA, Student’s t-test and Wilcoxon or Friedman tests were used.

**Results:**

Up to 30% of cardiac surgery patients had kidney failure at diagnosis of LCOS. Kidney failure at discharge from the ICU was more frequent in patients who received beta-agonist drugs as compared to those who received levosimendan (*p* < 0.05).

**Conclusion:**

The incidence of kidney failure decreased with the postoperative administration of levosimendan to cardiac surgery patients with LCOS, as compared to beta-agonists.

**Trial registration:**

Current Controlled Trials ISRCTN 46058317. Date of registration: 7/10/2019.

Retrospectively registered.

## Background

One of the most common and serious complications of cardiac surgery is the low cardiac output syndrome (LCOS), which incidence in cardiac surgery patients ranges from 15 to 25%. This syndrome is associated with a mortality of 15%, reaching 70% in patients who develop cardiogenic shock. LCOS also increases pulmonary, renal and cardiac morbidity [1].

Preoperative kidney failure is a predictor of perioperative risk in candidates to elective cardiac surgery [2]. Kidney failure is also one of the most relevant prognostic factors of mortality, and its incidence in patients with LCOS is 30 to 70%. Although kidney failure is reversible, patients who develop it have a higher risk of death than those who do not present this complication [3].

The gold standard treatment for LCOS is based on beta-agonist agents (adrenaline, dobutamine) [4]. Yet, calcium sensitizers such as levosimendan are used as an alternative therapy. Levosimendan is a calcium sensitizer that improves cardiac contractility and increases oxygen consumption, but to a lesser extent than other inotropes. Its mechanism of action is three-fold, namely: it is a positive inotropic, a vasodilator, and provides cardioprotection against myocardial ischemia and damage caused by ischemia-reperfusion [5]. Its active metabolite OR-1896 has similar properties and, as it forms slowly, the duration of its effects is at least 7 days. Several studies have analyzed its protective effects on postoperative renal function in cardiac surgery patients with LCOS [6]. The preoperative, intraoperative and postoperative effects of levosimendan have been assessed with conflicting results in terms of the timing of administration and type of patients. Bragadottir et al. associated levosimendan with improved glomerular filtration rates, renal flow and organ oxygen supply, which were independent from its cardioprotective effects [7]. Similar results were published by Ferreri et al. [8].

Our group published recently a series of articles demonstrating the protective effects of levosimendan on renal function in patients with severe ventricular dysfunction at high risk for kidney failure. These effects are partially exerted by drug-induced renal preconditioning via the opening of mitochondrial KATP channels [9].

The need to evaluate the possible nephroprotective effect of the drug in patients with low cardiac output syndrome, independent of its inotropic role is the objective of our study.

We postulated that the use of levosimendan as compared to beta-agonists in cardiac surgery patients with LCOS and kidney failure exerts beneficial preconditioning effects on renal function that are independent from its cardioprotective effects. We collected data from cardiac surgery patients who developed LCOS from the more than 600 patients who underwent cardiac surgery in our unit in the last 3 years. We investigated the potential protective role of levosimendan against renal dysfunction in these patients.

## Methods

A three-year study was performed (January 2015–May 2018) in the Virgen de la Victoria hospital, Malaga, Spain. It was a quasi-experiment study used to estimate the causal impact of an intervention on target population without random assignment. Quasi-experimental research shares similarities with the traditional experimental design or randomized controlled trial, but it specifically lacks the element of random assignment to treatment or control. As it was a quasi-experimental study, recruitment was maintained until 50 patients were reached in each of the groups; patient recruitment from each of the groups was completed upon reaching the number of patients determined for the sample size, requiring 1 month more in the case of patients treated with levosimendan to reach the number of 50 patients. As it was not a randomized study, the recruitment ended more quickly in those patients in whom the responsible physician decided to use beta agonists, in our case. Subsequently, the number of cases with patients with renal dysfunction in each of them was collected, which was 15 patients per group, there being no randomization criteria here.

### Ethics approval and consent to participate

Our study was both submitted to and approved by the ethics committee. Written Informed consent to participate in the study was obtained from participants.

The study was approved by the local Ethics Committee (Malaga Norte-Andalusian Public Foundation for Malaga Research in Biomedicine and Health).

Patients were divided into two groups based on the therapy received. Thus, patients were allocated either to receive beta-agonists or levosimendan at physician’s discretion. Inclusion and exclusion criteria were:

1. Patients older than 18 years who developed postoperative low cardiac output syndrome (LCOS) following heart surgery.
2. LCOS (was defined as: a cardiac index < 2 l/min/m^2^, or central venous saturation < 65% after volume replacement). It was checked by echocardiogram and Mostcare® (continuous cardiac index monitoring).
3. Patients who required inotropic support for the treatment of LCOS.

Exclusion criteria were:

1. Patients who required combined surgery (not only cardiac surgery)
2. Emergency surgery
3. Preoperative diagnosis of chronic kidney failure.

The administration of beta-agonists was maintained until LCOS resolution. The beta-agonists used for haemodynamic control were adrenaline or dobutamine, depending on whether vascular resistance concomitant to low cardiac output was low or high. Noradrenaline was added when necessary. Levosimendan was administered for 24 h at a rate of 0.1 mcg/kg/min to a target dose of 12.5 mg. The objectives of the therapy in the two groups were reaching a CI > 2 l/min/m^2^ and a central venous saturation > 65% following volume replacement.

Informed consent was obtained from all participants. The following data were collected from recruitment to ICU discharge: preoperative renal and cardiac function data (heart and renal function prior to surgery) at diagnosis of LCOS, at 24 h and 48 h after diagnosis (24 h following completion of levosimendan therapy) and at Intensive Care Unit (ICU) discharge.

- **Renal function parameters:** Creatinine, stage of kidney failure as measured by the Acute kidney failure (AKI) scale, diuresis and use of diuretics (mg of furosemide). Requirement of renal replacement therapy.
- **Hemodynamics:** Heart rate (HR), multifocal atrial tachycardia (MAT), heart failure (HF), and ejection fraction of the left ventricle (EFLV), as monitored by an ultrasound specialist.
- Central venous saturation (SvcO2), central venous pressure (CVP). Heart rate and Cardiac Index was continuously monitored using **MostCare**®.

Routine monitoring of all patients included continuous 5-lead ECG (leads II and V), invasive blood pressure in the radial artery, pulse oximetry and central venous saturation.

We estimated that a sample of 100 patients with LCOS was required, assuming a reported incidence of 30% of kidney failure in these patients for a 95% confidential interval (CI) and a minimum precision of 10% to compare the effects of levosimendan vs beta-agonists in patients with kidney failure at diagnosis. The team conducting the statistical analysis was blinded to the group analyzed. Epidat 4.0 was the statistical software used.

A descriptive statistical analysis was first conducted. Continuous variables were expressed in a table as means, standard deviations or medians based on normality of distribution. If continuous quantitative variables were normally distributed –as assessed by Shapiro-Wilk test– were measuerd by the ANOVA test. Differences between baseline values and values at 24 h and 48 h were assessed by Student’s t-test when normally distributed in each group. Normal distribution was tested by the Shapiro-Wilk test; otherwise, Wilcoxon or Friedman tests were used. The Wilcoxon and Friedman test are a non-parametric statistical hypothesis test used to compare two related samples, matched samples, or repeated measurements on a single sample to assess whether their population mean ranks differ (i.e. it is a paired difference test). It can be used as an alternative to the paired Student’s t-test (also known as “t-test for matched pairs” or “t-test for dependent samples”) when the population cannot be assumed to be normally distributed (Lowry, Richard. “Concepts & Applications of Inferential Statistics”).

Kidney failure was classified using the Acute Kidney Injury (AKI) scale according to variations in creatinine levels and diuresis.

## Results

In our study we finished the recruitment when 100 patients had a diagnosis of low cardiac output syndrome, these patients had an incidence of kidney failure at diagnosis of 30%. We studied both groups (levosimedan vs beta agonist), with 50 patients in each one Table 1.

**Table 1:** Epidemiological and surgical data from patients with postoperative low cardiac output syndrome

	Inotropic therapy			
	Beta-agonists (*n* = 50)		Levosimendan(*n* = 50)	
	%	Mean ± SD	Mean ± SD	%
Sex				
Female	25.0%			42.9%(*p* > 0.05)
Male	75.0%			57.1%(*p* > 0.05)
Total	100.0%			100.0%
Age				
Height		166.9 ± 7.8	165.5 ± 8.9	(*p* > 0.05)
Weight		76.5 ± 14.2	76.9 ± 13.8	(*p* > 0.05)
BMI - Category				
BMI – Category				
Underweight	0.0%			0.0%(*p* > 0.05)
Healthy weight	38.5%			22.2%(*p* > 0.05)
Overweight	38.5%			55.6%(*p* > 0.05)
Obesity	19.2%			22.2%(*p* > 0.05)
Morbid obesity	3.8%			0.0%(*p* > 0.05)
Total	100.0%			100.0%
EuroScore II (%)		2.7 ± 3. 3	3.2 ± 2.6	(*p* > 0.05)
NYHA				
II	46.2%			28.6%(*p* > 0.05)
III	28.8%			42.9%(*p* > 0.05)
IV	7.7%			10.7%(*p* > 0.05)
Total	100.0%			100.0%
Type of surgery				
Valve surgery	43.1%			33.3%(*p* > 0.05)
Coronary artery bypass with ECC	2.0%			7.4%(*p* > 0.05)
Coronary artery bypass without ECC	23.5%			40.7%(*p* > 0.05)
Combined	27.5%			18.5%(*p* > 0.05)
Ascending thoracic aortic	3.9%			0.0%
Total	100.0%			100.0%
Beta-blockers				
No	41.7%			30.0%(*p* > 0.05)
Yes	58.3%			70.0%(*p* > 0.05)
Total	100.0%			100.0%(*p* > 0.05)
ACEs				
No	55.6%			60.0%(*p* > 0.05)
Yes	44.4%			40.0%(*p* > 0.05)
Total	100.0%			100.0%(*p* > 0.05)
ARBs II				
No	63.9%			75.0%(*p* > 0.05)
Yes	36.1%			25.0%(*p* > 0.05)
Total	100.0%			100.0%(*p* > 0.05)
Clamping ischemia (minutes)		72.9 ±	67.67 ±	(*p* > 0.05)
Therapy for dyslipidemia				
No	7.7%	31.31	37.9	6.3%(*p* > 0.05)
Yes	92.3%			93.8%(*p* > 0.05)
Total	100.0%			100.0%

There were no significant differences across groups in any of the parameters analyzed (*p* > 0.05)

*BMI* Body mass index

*ACEs* Angiotensin-converting enzyme

*ARBs* II Angiotensin II Receptor blockers

*SD* Standard deviation

Thirty patients developed kidney failure following cardiac surgery. Kidney failure persisted at discharge (as assessed by the AKI scale) only in some patients in the beta-agonist group, with significant differences between the two treatment groups (*p* < 0.05). None of the patients required renal replacement therapy.

Diuresis at diagnosis of LCOS in patients who were treated with beta-agonists and did not develop kidney failure was 0.89+/− 0.38 ml/kg/h 24 h. before diagnosis, and 1.09+/− 0.41 ml/kg/h at 48 h. Creatinine was 1.24 +/− 1.68 mg/dl at baseline; 1.78 +/− 4.57 mg/dl at diagnosis of LCOS; 1.62 mg/dl+/− 3.21 mg/dl at 24 h; 1.56 mg/dl+/− 3.71 mg/dl at 48 h; and 0.97 mg/dl+/− 0.94 mg/dl at discharge Table 2.

**Table 2:** Differences in creatinine levels and diuresis between patients treated with beta-agonists + levosimendan for low cardiac output syndrome who exhibited kidney failure at diagnosis and evolution of creatinine and diuresis at 24 h, 48 h, and at discharge from the ICU

Inotropic therapy			
	Beta-agonists (*n* = 15)		Levosimendan (n = 15)
	No Kidney failure at Discharge (*n* = 9)	Kidney failure at Discharge (*n* = 6)	No Kidney failure at Discharge (*n* = 15)
	Mean +/−SD	Mean +/−SD	Mean +/−SD
Diuresis 24 h before diagnosis of LCOS (ml/kg/h)	0.89+/− 0.38	0.85+/− 0.38	1.66+/−0.38
Diuresis at 24 h. (ml/kg/h)	0.91 +/− 0.30	0.64 +/− 0.23	1.83 +/5.92
Diuresis 48 h. after diagnosis of LCOS (ml/kg/h)	1.09+/− 0.41	0.82 +/− 0.32	2.10+/− 6.26 (*p* < 0.05)
Baseline creatinine (mg/dl)	1.24 +/−1.68	1.25 +/− 0.82	1.09 +/− 0.82
Creatinine at diagnosis of LCOS (mg/dl)	1.78 +/− 4.57	1.52 +/− 2.56	1.87 +/− 0.24
Creatinine 24 h after diagnosis of LCOS (mg/dl)	1.62 +/− 1.68	2.1 +/−1	1.32+/−3.5
Creatinine 48 h after diagnosis of LCOS (mg/dl)	1.56+/− 3.71	2.72+/− 1*	1.22 +/− 0.53 (p < 0.05)
creatinine levels (mg/dl)	0.97+/− 0.44	2.57 +/− 0.72*	1.21+/− 0.74(*p* < 0.05)

The patients who were administered levosimendan did not develop kidney failure. In contrast, the incidence of kidney failure at discharge in patients who had renal dysfunction at diagnosis of LCOS who received beta-agonists was 40%

*LCOS* low cardiac output syndrome

Diuresis at diagnosis of LCOS in patients who were treated with beta-agonists and developed kidney failure was 0.85+/− 0.38 ml/kg/h 24 h. prior to diagnosis, and 0.82 +/− 0.32 ml/kg/h at 48 h of treatment. Creatinine was 1.25 +/− 0.82 mg/dl at baseline; 1.52 +/− 2.56 mg/dl at diagnosis of LCOS; 2.1 mg/dl +/− 0.5 mg/dl at 24 h; 2.57 mg/dl+/− 0.72 mg/dl at 48 h; and 2.57 mg/dl+/− 0.72 mg/dl at discharge Table 2.

Diuresis at diagnosis of LCOS in patients who were treated with levosimendan was 1.66+/− 0.38 ml/kg/h 24 h. prior to diagnosis, and 2.10+/− 6.26 ml/kg/h at 48 h of treatment. Creatinine was 1.09 +/− 0.82 mg/dl at baseline; 1.87 +/− 0.24 mg/dl at diagnosis of LCOS; 1.32 mg/dl +/− 3.5 mg/dl at 24 h; 1.22 mg/dl+/− 0.53 mg/dl at 48 h; and 1.21+/− 0.74 mg/dl at discharge. Significant differences were observed in creatinine levels, diuresis and AKI scores between the levosimendan group and the other two groups at 48 h, and between the levosimendan group and the group of patients who developed kidney failure at discharge (*p* < 0.05) (Table 2).

In the levosimendan group, we investigated whether a relationship existed between the timing of administration (intra vs postoperative) and kidney failure, but no significant differences were found. Yet, a tendency was observed in the incidence of kidney failure to be lower in the group that was administered levosimendan intraoperatively vs postoperatively (*p* < 0.05).

There were no significant differences between groups in the use of furosemide (only diuretic used in our study) (*p* > 0.05).

There were no significant differences between groups in haemodynamics at diagnosis or at 24 h after completion of levosimendan therapy (*p* > 0.05) Table 3.

**Table 3:** Haemodynamic parameters for the patients who developed LCOS in the two study groups

	Beta-agonists (*n* = 50)	Levosimendan (*n* = 50)
	Mean ± SD	Mean ± SD
Heart rate (beats / minute) (at diagnosis of Low Cardiac Output Syndrome)	78.7 ± 16.2	79.8 ± 12.4
Heart rate (beats / minute) (at 48 h. of treatment)	83.9 ± 16.2	82.82 ± 20.9
Mean blood pressure (mmHg) (at diagnosis of Low Cardiac Output Syndrome)	68.7 ± 11.11	67.5 ± 10.3
Mean blood pressure (mmHg) (at 48 h of treatment)	75.6 ± 9.3	70.4 ± 13.2
Central venous pressure (mmHg) (at diagnosis of Low Cardiac Output Syndrome)	9.7 ± 4.6	8.7 ± 3.6
Central venous pressure (mmHg) (at 48 h of treatment)	10.1 ± 3. 3	10.7 ± 2.2
Cardiac index (l/min/m2) (at diagnosis of Low Cardiac Output Syndrome)	2.4 ± 0.7	2.1 ± 0.4
Cardiac index (l/min/m2) (at 48 h. of treatment)	2.6 ± 0.4	2.9 ± 17.6
SatVO2 (%) (at diagnosis of Low Cardiac Output Syndrome)	65.3 ± 9.9	65.9 ± 10.3
SatVO2 (%) (at 48 h of treatment)	71.71 ± 9. 9	64.7 ± 12.5

There were no haemodynamic differences between groups at diagnosis or at 24 h of completion of levosimendan therapy (48 h of treatment)

*ns* not significant (*p* > 0.05)

No statistically significant differences were observed in ICU length of stay (levosimendan 4 days+/− 2 days vs beta agonists 5 days +/− 3 days, p > 0.05); although sample size was not calculated for this variable.

## Discussion

The most common and serious postoperative complications identified were low cardiac output syndrome and kidney failure. Perioperative cardioprotective and renoprotective strategies consist of maintaining organ perfusion and oxygen supply to tissues. Appart from diuretics, renal replacement therapy is another therapeutic option for acute kidney injury. When response to inotropic support is not adequate, intra-aortic balloon pump (IABP) counterpulsation and other ventricular assist devices can be used. However, none of our patients required any of these extracorporeal therapies [10]. The effect of levosimendan could be two-fold: first, its inotropic effects may improve cardiac output. Secondly, in a setting of low cardiac output, levosimendan has a direct effect on renal perfusion, as it is a KATP channel opener with a vasodilator effect on arteries. In addition, in a setting of renal hypoperfusion due to decreased cardiac output, levosimendan provides kidney protection by blocking mitochondrial KATP channels.

According to the literature, the incidence of kidney failure in LCOS is 20 to 30%, which is consistent with our results.

The drug used for treating LCOS was an independent factor of kidney failure. In our study, the incidence of kidney failure at 48 h after treatment completion was lower in the patients who were administered the calcium sensitizer for LCOS, as compared to those who received beta-agonists. Similar results have been obtained in previous comparative studies of levosimendan and beta-agonists or placebo. Levin et al. performed a study in 252 patients with severe left ventricular dysfunction undergoing coronary artery bypass grafting with cardiopulmonary bypass who developed LCOS. Preoperative levosimendan was compared with placebo, and the incidence of kidney failure was found to be lower with levosimendan [11]. Baysal et al. randomized 128 patients with FEVI < 45% undergoing mitral valve surgery to two treatment arms. The first group received a loading dose of 6 mcg/kg/min of levosimendan after removal of the aortic cross-clamp followed by an infusion of 0.1mcg/kg/min in combination or not with standard inotropics (dobutamine, adrenaline and noradrenaline). The control group only received standard inotropics. Postoperative serum creatinine levels were lower in the first group, who also showed a better glomerular filtration rate [12]. In contrast with previous works, our study focused on cardiac surgery patients who developed organ dysfunction caused by hypoperfusion induced by postoperative LCOS.

Differences were also observed in the incidence of kidney failure –not significant but with a clear tendency– based on the timing of levosimendan administration. Early administration and the agent selected were found to be determinant. Differences may have not reached significance because our sample size was not calculated for this endpoint.

Balzer et al. conducted a retrospective study in 46 patients who underwent coronary artery bypass grafting with creatinine < 2 mg/dl and a left ventricular systolic function < 35% and/or LOCS. The incidence of acute kidney failure was significantly lower in patients who received early levosimendan therapy vs. those who received late-start levosimendan therapy [13]. Treskatsch et al. performed a study in 159 cardiac surgery patients with FEVI < 35% and/or LCOS, who were divided into two groups based on the timing of levosimendan administration. Early administration was found to be related to a lower incidence of kidney failure and a reduced need for renal replacement therapy [14]. In a multicenter, randomized, double-blind, placebo-controlled LICORN study, Caruba et al. included 340 patients from 13 hospitals with FEVI < 40% undergoing CABG with cardiopulmonary bypass. The authors investigated the effects of levosimendan administered prior to anesthesia vs placebo and the requirement of renal replacement therapy while in the ICU. A lower incidence was demonstrated in the levosimendan group [15]. In our study, the incidence of kidney failure at 24 h showed a decreasing tendency following early administration of levosimendan vs beta-agonists at diagnosis of LCOS, with differences becoming significant at 48 h of diagnosis. Sample homogeneity in terms of cardiac function and oxygen supply was ensured by measuring central venous saturation and cardiac index, guaranteeing that the hemodynamic therapy administered for LCOS was similar in the two groups. All LCOS patients received inotropic therapy, and their cardiac index and venous saturation were within normal limits. The incidence of renal dysfunction was lower in the levosimendan group. This finding supports our hypothesis that the protective effect of levosimendan on renal function is independent from its cardioprotective effect. Bragadottir et al. documented that levosimendan has beneficial effects on renal blood flow, glomerular filtration rate and renal vascular resistance, as a result of its action on KATP channels in renal afferent arterioles [7]. Levosimendan improves renal function (defined as a reduction in serum creatinine) at 24 h after therapy completion and enhances diuresis at ICU discharge by reducing serum creatinine levels.

Although our results are consistent with those obtained by other authors such as Fedele et al., who documented the benefits of levosimendan on renal function [16], conflicting results have been obtained in other studies. Thus, Mehta et al. examined data from 849 patients with severely reduced ejection fraction who underwent cardiac surgery requiring cardiopulmonary bypass support (66% had coronary artery bypass surgery). With a third of patients having mild preoperative chronic kidney failure, no significant differences were found in the need for renal replacement therapy at 30 days between patients who received preoperative levosimendan vs placebo [17]. Similar results were reported by Landoni in a multicenter, randomized, double-blind study in 506 patients with perioperative ventricular dysfunction (preoperative FEVI < 25%; need for IABP or elevated postoperative score). In this study, most patients were recruited in the postoperative period and presented LCOS requiring hemodynamic support with high doses of inotropics that could be secondary to LCOS-induced cardiogenic shock. No differences have been reported either between levosimendan and placebo in the incidence of kidney failure as measured by the AKI scale, or in the need for renal therapy [18]. Inconsistencies could be due to the population of patients. In our group, patients prevailingly had mitral and mitral-tricuspid dysfunction, where the incidence of postoperative right ventricular dysfunction is higher. Also, the sample did not include any patient with cardiogenic shock. In a previous study, our group evaluated a case series of patients who underwent valve replacement/ repair surgery, who were administered levosimendan preoperatively. The hemodynamics of patients with preoperative right ventricular dysfunction improved as a result of improved postoperative right ventricular and renal function [19]. As described by Damman et al., an elevated renal venous pressure causes a reduction in perfusion pressure and renal blood flow. When ventricular function improves, renal venous pressure decreases, thereby exerting a renoprotective effect [20]. In two studies previously published by our group, preoperative levosimendan was found to protect renal function in patients at high risk for cardio-renal dysfunction, as assessed by the AKI scale and postoperative N-GAL levels. Thus, the results of our study may be explained by both, the potential renoprotective effects of levosimendan and the underlying heart disease of our patients, whose biventricular –and, especially, right ventricular– function improved.

The possibilities of a relationship between the effect on potassium channels, as well as the possible properties related to pharmacological post-conditioning, in addition to the improvement of renal perfusion through the treatment of low cardiac output, should be the starting point again clinical trials that have the ability to confirm our findings.

### Limitations

The main limitation of this study was that patients were not randomized to treatment groups. The reason is that we preferred that physicians treated their patients with the medications they were more familiar with. This may have caused bias in the interpretation of results, as physicians will probably favor the conventional therapy. A triple-blinded study was not possible due to the type of study. However, the team conducting statistical analysis was blinded to the group analyzed. The use of certain biochemical parameters with greater sensitivity and specificity (N-GAL or Cystatin C), would also be useful to evaluate their action.

## Conclusion

The incidence of kidney failure decreased with the postoperative administration of levosimendan to cardiac surgery patients with LCOS, as compared to beta-agonists.

## Data Availability

The datasets used and/or analysed during the current study are available from the corresponding author on reasonable request.

## Abbreviations

AKI: Acute kidney failure
CI: Confidential interval
CVP: Central venous pressure
EFLV: Ejection fraction of the left ventricle
HF: Heart failure
HR: Heart rate
IABP: Intra-aortic balloon pump
ICU: Intensive Care Unit
LCOS: Low cardiac output syndrome
MAT: Multifocal atrial tachycardia
SvcO2: Central venous saturation
